# Effect of Botulinum Toxin Injection on Asymmetric Lower Face with Chin Deviation

**DOI:** 10.3390/toxins12070456

**Published:** 2020-07-17

**Authors:** Dongwook Kim, Ju-Hyun Park, Vittorio Favero, James Mah, Young-Soo Jung, Seong Taek Kim

**Affiliations:** 1 Department of Oral & Maxillofacial Surgery, Yonsei University College of Dentistry, Seoul 03722, Korea; dwkimomfs@yuhs.ac; 2 Department of Orofacial Pain & Oral Medicine, Yonsei University College of Dentistry, Seoul 03722, Korea; cookie205@naver.com; 3 Section of Dentistry and Maxillofacial Surgery, Department of Surgery, University of Verona, 37129 Verona, Italy; vittorio_favero@yahoo.it; 4 Advanced Education Program in Orthodontics and Dentofacial Orthopedics, School of Dental Medicine, University of Nevada, Las Vegas, NV 89154, USA; drjamesmah@gmail.com

**Keywords:** botulinum toxins, asymmetry, posteroanterior (PA) cephalometrics

## Abstract

The purpose of this study was to compare the efficacy of botulinum toxin (BoNT) in masseter muscle reduction depending on the amount of chin deviation. Exploring distinctive effects of BoNT relative to the characteristics of facial asymmetry will aid in planning and predicting treatment outcomes. Sixteen adult volunteers were classified into two groups according to the degree of menton deviation observed in posteroanterior cephalograms. Eight had a menton deviation of 3 mm or more and the other eight had less than 3 mm. A total of 25 Units of BoNT was injected into the unilateral masseter muscle of the prominent side for each participant. Changes in the volume and bulkiest height of the lower face on each side were measured with a 3D laser scan at four time points: before and 4, 8, and 12 weeks after the injection. Two-way mixed ANOVA was employed for analyses. The volume and bulkiest height of the injected side decreased over time in both types of asymmetry, with significant differences at each time point. The reductions in the volume and bulkiest height were significantly greater in subjects without chin deviation. The reductions in the volume and bulkiest height of the lower face using BoNT are more effective for subjects without chin deviation.

## 1. Introduction

There has been growing demand for the correction of facial asymmetry as patient expectations have increased [1]. Disharmony of lower facial width is a type of facial asymmetry whose mainstay corrective surgical procedures include orthognathic surgery, resection of the corresponding portion of the mandible, and resection of the masseter muscle [1,2]. Meanwhile, patients tend to prefer less invasive options, such as botulinum neurotoxin (BoNT) injection into masseter muscle to reduce its volume [3].

The BoNT is produced by *Clostridium botulinum* and consists of a heavy chain and a light chain linked together by a single disulphide bond [4]. It is synthesised as a relatively inactive single-chain polypeptide approximately 150 kDa, and is activated when proteolytically cleaved into the 100-kDa heavy chain and the 50-kDa light chain [5]. While BoNT exists in seven different serotypes, named A, B, C, D, E, F and G, type A has been the most widely used for therapeutic purposes. The BoNT permanently binds to the motor end plate at the neuromuscular junction which prevents the release of acetylcholine from the pre-synaptic vesicles causing a pre-synaptic blockade [3].

BoNT injection is a meritorious method for improving facial esthetics. The chemodenervation, via BoNT injection, can improve facial wrinkles and reduce the width of the lower face by means of masstere muscle atrophy [6]. Due to its synergestic effects, it has been applied to various procedures aimed at enhancing facial esthetics [6]. The first report of BoNT for masseter muscle hypertrophy was in 1994. Local injection of BoNT into a muscle produces paralysis and atrophy [7]. Side effects and possible reactions of BoNT injection into masseter muscle are minor, including temporary paralysis of adjacent facial muscles, soreness and swelling at the injection site, which resolves within 2 weeks [8].

As precisely predicting the post-treatment outcome in facial asymmetry remains challenging, given the diverse patterns of asymmetry and the involvement of both skeletal and soft tissue changes, the asymmetry can remain after the corrective surgery [1,2,9]. BoNT can be utilized as an adjunct procedure in such cases. However, the efficacy of BoNT may vary according to the character and severity of the asymmetry.

The purpose of this study was to compare the efficacy of BoNT injection into masseter muscle depending on the amount of chin deviation, a critical element in the perception of facial asymmetry [10].

## 2. Results

### 2.1. Demographic Data

There was no statistically significant difference between the demographic data of the two groups other than the amount of menton deviation (Table 1).

### 2.2. Summary Statistics

The means and standard deviations of the height and volume change compared to the pre-injection state at each time point were acquired (Table 2).

### 2.3. Assumption Check

The height and volume change were normally distributed for each combination of factor levels as assessed by Shapiro–Wilk’s test of normality (*p* > 0.05). There was homogeneity of variances as assessed by Levene’s test (*p* > 0.05). The homogeneity of covariances was present as assessed by Box’s test of equality of covariance matrices (*p* > 0.001).

### 2.4. Report

**Figure 1:**
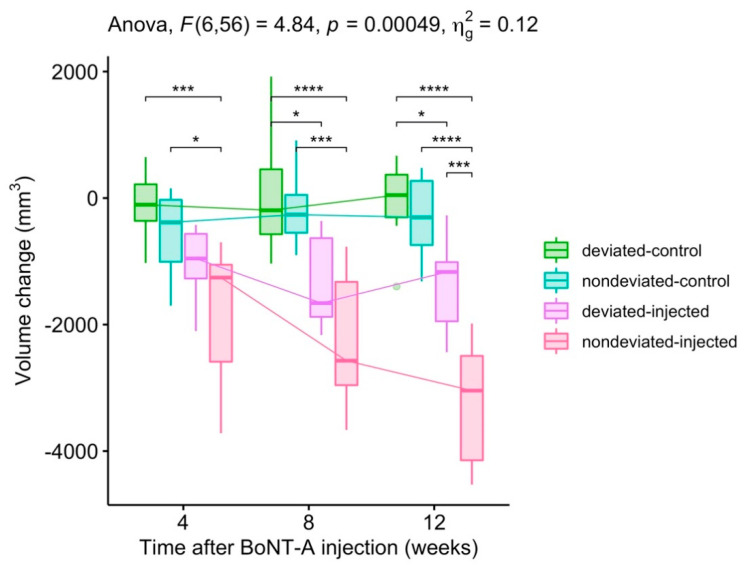
Changes in the volume of the lower face. Pairwise comparison: *T*-test; Adjustment method: Bonferroni correction (* *p* < 0.05, ** *p* < 0.01, *** *p* < 0.001, **** *p* < 0.0001).

**Figure 2:**
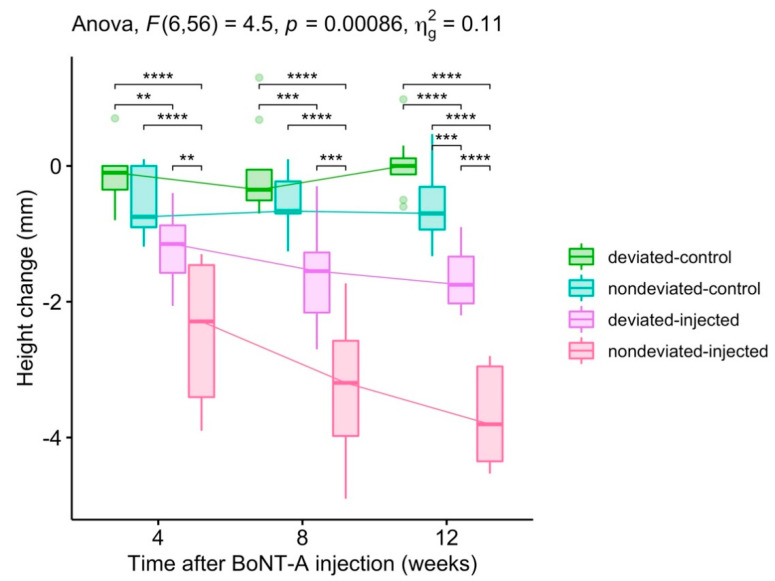
Changes in the bulkiest height the of lower face. Pairwise comparison: *T*-test; Adjustment method: Bonferroni correction (* *p* < 0.05, ** *p* < 0.01, *** *p* < 0.001, **** *p* < 0.0001).

There was a statistically significant two-way interaction between the groups and time in explaining both the volume and height change. Volume: F (6, 56) = 4.835, *p* < 0.001 (Figure 1). Height: F (6, 56) = 4.505, *p* < 0.001 (Figure 2).

### 2.5. Post-Hoc Tests

In terms of volume change, the simple main effect of the groups was significant at all time points considering the Bonferroni-adjusted *p*-value (*p* < 0.0001) (Table 3). This was also the case in the bulkiest height change.

Pairwise comparisons show that the mean volume change was significantly different between the control side and injected side for each group of chin deviation at 8 and 12 weeks. The comparison within injected sides between deviated and non-deviated chin groups revealed statistically significant differences at 12 weeks (Table 4).

Similar, but more prominent, pairwise differences were noted among bulkiest height changes. Significant difference was present between most of the groups at all time points except between controls and a few groups at 4 and 8 weeks. The bulkiest height change was significantly different within all groups at 12 weeks (Table 5).

### 2.6. Summary

In brief, the volume and bulkiest height on the BoNT injected side decreased over time in both types of asymmetry. The reductions in volume and bulkiest height by BoNT were significantly greater in the non-deviated chin group than in the group with chin deviation (Figure 1 and Figure 2).

## 3. Discussion

The etiology of each type of facial asymmetry may differ, each requiring appropriate plans for optimal outcomes. Likewise, it can be assumed that the effect of BoNT may differ according to the facial asymmetry characteristics. The etiology of a deviated chin is believed to be asymmetric growth of the condyle or the entire mandible [11]. Given the several analyses and classifications of facial asymmetry based on different etiologic factors, it can be assumed that the effect of BoNT may vary depending on the etiology of the facial asymmetry [1,12].

Several tools are used for the diagnosis and classification of facial asymmetry. Posteroanterior (PA) cephalography provides information about diagnostic structures and is easy to perform while also being low-cost. For these reasons, PA cephalography has long been useful for diagnosis and planning; even in the era of 3D analysis, its reliability and validity has been well-established [1,13,14].

The skeletal midsagittal reference line (MSR) was established from the crista galli, vertically through the anterior nasal spine and extended inferiorly beneath the chin; this method is known to be highly reproducible [13]. Deviation of the menton in particular, which is located at the most inferior region of the mandible, is the most likely cause of perceived facial asymmetry [10,15]. We thus selected the menton as a reference point for measurements of facial asymmetry and then classified the findings into two types according to the degree of deviation of the menton from the MSR based on PA cephalograms. Approximately 3 to 4 mm of deviation is reported to be the perceivable threshold and is thus considered clinically significant [16].

There has been a steady demand for the treatment of facial asymmetries in even the mildest amount. Demand for non-surgical or less invasive means also exists. BoNT therapy is a relatively simple and aesthetically effective treatment for facial asymmetry by means of reducing the volume of the masseter muscle [8]. Previous studies have reported the efficacy of BoNT for facial asymmetry. Shim et al. reported the volume and width of the lower face decreases significantly after 4, 8, 12, and 24 weeks [3]. Kim et al. reported that the majority patients who underwent BoNT injection for the purpose of lower face contouring were satisfied with the results [17]. Since its introduction for cosmetic use in 1994, specifically for the treatment of masseteric hypertrophy, there have been many clinical studies into the efficacy of BoNT treatment using photography, ultrasonography, and computed tomography (CT) as analysis tools [3].

However, recently developed tools such as three-dimensional (3D) laser scanning are now being used to evaluate the efficacy of BoNT, since they allow for the accurate measurement of changes in the external facial contours [18]. The technology also allows the change in facial appearance to be readily demonstrated to both patients and clinicians. The laser scan provides more accurate measurements of the facial contours compared to other analysis tools [18,19].

As facial asymmetry itself is a three-dimensional matter, a two-dimensional measure may be insufficient when analyzing it with PA cephalograms. However, even defining the midline of face and facial asymmetry itself becomes more complex in a three-dimensional setting, as witnessed by the increasing number of studies published over recent decades [1,14,15,20,21,22]. Considering that the perception of asymmetry is subjective rather than analytic in a real-life setting, the powerful criteria of menton deviation in a two-dimensional cephalogram may still be useful for research purposes [20]. In this study, the changes in volume and bulkiest height were measured by 3D laser scanning, and differences obtained by subtracting after superimposition yielded accurate results.

After the onset of BoNT-induced muscle atrophy, new neuromuscular synapses may form as a result of sprouting of pre-synaptic axons, resulting in muscle recovery [7]. A better understanding of the effect of BoNT will require follow-up of any resulting change in the volume and bulkiest height for a minimum of 12 months. In addition, the degree of recovery may differ according to the type of facial asymmetry.

While lateral changes following orthognathic surgery are relatively easy to predict, precise prediction of postoperative symmetry remains challenging, despite patient expectation [1]. Soft-tissue thickness is known to camouflage hard-tissue asymmetry, so the actual skeletal asymmetry may be greater than that seen in the patient’s face [1]. Similarly, soft-tissue change is known to compensate for hard-tissue changes in the frontal plane, which may make the quantitative prediction of soft tissue changes more difficult, resulting in post-surgery asymmetry [23]. In such cases, this study suggests that BoNT might be considered as an adjunctive measure to reduce the more prominent side for the resolution of transverse asymmetry of the lower face.

## 4. Conclusions

BoNT significantly reduced the soft-tissue volume and height of the lower face over a 12 week time period. The reduction was significantly more effective in cases without chin deviation. Therefore, BoNT injection can be considered for the resolution of transverse asymmetry of the lower face by means of reducing the prominent side, especially in cases without chin deviation.

## 5. Materials and Methods

### 5.1. Patients

Sixteen volunteers aged 20 and older, complaining of an asymmetric lower face, were enrolled in this study. Exclusion criteria were pregnancy, history of drug allergy, and any other systemic illnesses. Informed consent was obtained from all of the volunteers, who were advised of their freedom to withdraw from the study at any time. The study was approved by the Yonsei University Dental Hospital Institutional Review Board (IRB No. 2-2015-0037) on 17 September 2015.

### 5.2. Facial Asymmetry and Group

**Figure 3:**
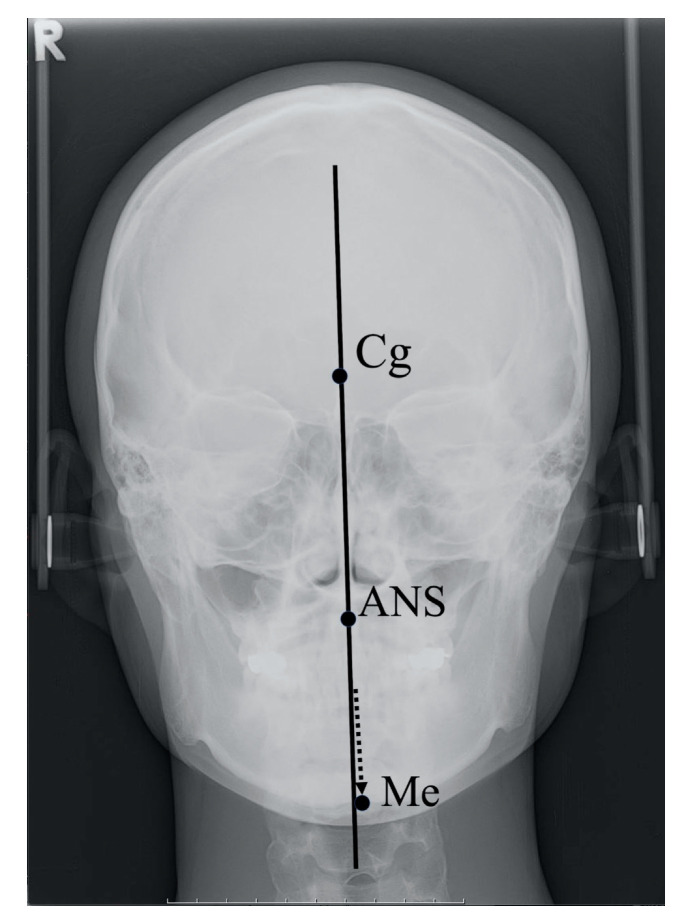
Landmarks used to determine the midsagittal reference line and menton deviation in posteroanterior cephalography. Soft tissue image superimposed to aid understanding. Arrow indicates the location of menton. Cg, crista galli; ANS, anterior nasal spine; Me, menton.

The facial asymmetry of each patient was assessed using posteroanterior (PA) cephalography taken before the injection in all subjects. The skeletal midsagittal reference (MSR) line was established from the crista galli (Cg), vertically through the anterior nasal spine (ANS) and extended inferiorly beneath the chin; this method is known to be highly reproducible [13]. Deviation of the menton (Me), the most inferior point of the chin, is thought to be the lead cause of perceived facial asymmetry [15] (Figure 3).

Patients were divided into two groups based on the extent of ME deviation. The first group was with a deviated chin, defined by Me deviation of 3 mm or more. The other showed Me deviation of less than 3 mm, below the threshold of clinical significance and thus considered a non-deviated chin [16].

### 5.3. BoNT Injection

A total of 25 U of BoNT type A was injected into the unilateral masseter muscle of the prominent side for each participant using a 1 mL syringe with a 29-gauge, 1/2-inch-long needle. The BoNT was injected into two points 1 cm apart at the center of the lower one-third of the identified masseter muscle, which is known to be the safest and most efficient injection site for BoNT. The uninjected contralateral side was assigned as a control for each group.

### 5.4. Measurement

The effect of BoNT was evaluated by measuring the changes in the volume and bulkiest height of the lower face on each side with a 3D laser scan using the Vivid 9i laser scanner (Minolta, Tokyo, Japan). The 3D laser scan was conducted at four time points: before the injection and 4, 8, and 12 weeks thereafter. A single technical expert performed all the scans, and all images were merged into single 3D facial images using image analysis software (Rapidform 2004, Inus Technology, Seoul, Korea). The border of the lower face was delineated using the following reference points: ala; cheilion; labral inferior; soft-tissue pogonion; soft-tissue menton; soft-tissue gonion and tragion. Differences in the volume and bulkiest height of the lower face were measured by superimposition of the data obtained over time.

### 5.5. Statistical Analyses

Mann–Whitney U test and Fisher’s exact test was used for comparing demographic factors of study objects. A two-way mixed measures analysis of variance (ANOVA) with Bonferroni correction was used to compare the means of three groups cross-classified by two independent variables: a between-subjects factor, group, and a within-subjects factor, time. Post-hoc tests were done using one-way ANOVA for simple main effect and pairwise independent t-tests for simple pairwise comparisons. Statistical significance was considered at the level of 0.05. Statistical analyses were performed using the R programming language (R Core Team, Vienna, Austria, 2020) [24].

## Figures and Tables

**Table 1:** Demographic data of the subjects.

Variables	Deviated Chin	Non-Deviated Chin	*p*	
	(*n* = 8)	(*n* = 8)		
Age [IQR]	25.0 [22.0; 33.0]	26.5 [25.0; 34.0]	0.311	Mann–Whitney U test
Sex			1.000	Fisher’s Exact Test
Female	5 (62.5%)	6 (75.0%)		
Male	3 (37.5%)	2 (25.0%)		
Menton deviation (mean ± SD)	5.4 ± 1.2	1.5 ± 0.9	0.002	*t*-test

IQR: Interquartile range, SD: Standard deviation.

**Table 2:** Mean change in the volume and bulkiest height of the lower face at each time point. A minus sign indicates a reduction in measurement.

		BoNT Injection	Time after BoNT Injection					
			4 Weeks		8 Weeks		12 Weeks	
Δ Volume (mm^3^) mean (SD)	Group I deviated chin	control	−76	(551)	112	(1105)	−59	(660)
		injected	−1015	(561)	−1364	(715)	−1389	(735)
	Group II non-deviated chin	control	−572	(679)	−180	(566)	−323	(691)
		injected	−1783	(1126)	−2266	(1029)	−3242	(994)
Δ Bulkiest height (mm) mean (SD)	Group I deviated chin	control	−0.14	(0.44)	−0.09	(0.7)	0.03	(0.49)
		injected	−1.22	(0.55)	−1.62	(0.74)	−1.66	(0.48)
	Group II non-deviated chin	control	−0.56	(0.51)	−0.53	(0.44)	−0.58	(0.59)
		injected	−2.45	(1.06)	−3.26	(1.09)	−3.70	(0.74)

BoNT: Botulinum toxin, SD: Standard deviation.

**Table 3:** Effect of group in bulkiest height and volume change at each time point. ANOVA.

Change	Time	Effect	Bonferroni-Adjusted *p*-Value	
Volume change	4 weeks	Group	3.00 × 10^−3^	**
	8 weeks	Group	7.20 × 10^−5^	****
	12 weeks	Group	7.46 × 10^−8^	****
Bulkiest height change	4 weeks	Group	4.71 × 10^−6^	****
	8 weeks	Group	7.65 × 10^−8^	****
	12 weeks	Group	4.32 × 10^−12^	****

(* *p* < 0.05, ** *p* < 0.01, *** *p* < 0.001, **** *p* < 0.0001).

**Table 4:** Pairwise comparisons between group levels for volume change. Pairwise *t*-test.

Time	Group1	Group2	Bonferroni-Adjusted *p*-Value		
4 weeks	deviated-control	nondeviated-control	1.00		
4 weeks	deviated-control	deviated-injected	1.24 × 10^−1^		
4 weeks	deviated-control	nondeviated-injected	7.34 × 10^−4^	***	
4 weeks	nondeviated-control	deviated-injected	1.00		
4 weeks	nondeviated-control	nondeviated-injected	2.26 × 10^−2^	*	
4 weeks	deviated-injected	nondeviated-injected	3.29 × 10^−1^		
8 weeks	deviated-control	nondeviated-control	1.00		
8 weeks	deviated-control	deviated-injected	1.40 × 10^−2^	*	
8 weeks	deviated-control	nondeviated-injected	5.67 × 10^−5^	****	
8 weeks	nondeviated-control	deviated-injected	7.21 × 10^−2^		
8 weeks	nondeviated-control	nondeviated-injected	3.45 × 10^−4^	***	
8 weeks	deviated-injected	nondeviated-injected	3.01 × 10^−1^		
12 weeks	deviated-control	nondeviated-control	1.00		
12 weeks	deviated-control	deviated-injected	1.21 × 10^−2^	*	
12 weeks	deviated-control	nondeviated-injected	4.29 × 10^−8^	****	
12 weeks	nondeviated-control	deviated-injected	6.51 × 10^−2^		
12 weeks	nondeviated-control	nondeviated-injected	2.32 × 10^−7^	****	
12 weeks	deviated-injected	nondeviated-injected	3.35 × 10^−4^	***	

(* *p* < 0.05, ** *p* < 0.01, *** *p* < 0.001, **** *p* < 0.0001).

**Table 5:** Pairwise comparisons between group levels for bulkiest height change. Pairwise t-test.

Time	Group1	Group2	Bonferroni-Adjusted *p*-Value	
4 weeks	deviated-control	nondeviated-control	1.00	
4 weeks	deviated-control	deviated-injected	2.30 × 10^−2^	*
4 weeks	deviated-control	nondeviated-injected	1.62 × 10^−6^	****
4 weeks	nondeviated-control	deviated-injected	3.83 × 10^−1^	
4 weeks	nondeviated-control	nondeviated-injected	4.29 × 10^−5^	****
4 weeks	deviated-injected	nondeviated-injected	7.93 × 10^−3^	**
8 weeks	deviated-control	nondeviated-control	1.00	
8 weeks	deviated-control	deviated-injected	2.99 × 10^−3^	**
8 weeks	deviated-control	nondeviated-injected	4.23 × 10^−8^	****
8 weeks	nondeviated-control	deviated-injected	5.16 × 10^−2^	
8 weeks	nondeviated-control	nondeviated-injected	6.94 × 10^−7^	****
8 weeks	deviated-injected	nondeviated-injected	1.40 × 10^−3^	**
12 weeks	deviated-control	nondeviated-control	2.83 × 10^−1^	
12 weeks	deviated-control	deviated-injected	1.97 × 10^−5^	****
12 weeks	deviated-control	nondeviated-injected	2.01 × 10^−12^	****
12 weeks	nondeviated-control	deviated-injected	5.49 × 10^−3^	**
12 weeks	nondeviated-control	nondeviated-injected	1.27 × 10^−10^	****
12 weeks	deviated-injected	nondeviated-injected	7.98 × 10^−07^	****

(* *p* < 0.05, ** *p* < 0.01, *** *p* < 0.001, **** *p* < 0.0001).
